# “OPERATION PHALCO”—Adapted Physical Activity for Breast Cancer Survivors: Is It Time for a Multidisciplinary Approach?

**DOI:** 10.3390/cancers15010034

**Published:** 2022-12-21

**Authors:** Arianna Murri, Daniela Vitucci, Eliana Tranchita, Elisa Grazioli, Stefania Gori, Alessandra Modena, Monica Turazza, Roberto Filippini, Silvia Galeazzi, Matteo Verzè, Patrizia Frittelli, Domenico Cristiano Corsi, Fabrizio Nicolis, Attilio Parisi, Claudia Cerulli

**Affiliations:** 1 Department of Movement, Human and Health Science, University of Rome «Foro Italico», 00135 Rome, Italy; 2 Department of Movement Science and Wellness, University Parthenope, 80133 Naples, Italy; 3 CEINGE Biotecnologie Avanzate Franco Salvatore, 80131 Naples, Italy; 4 Medical Oncology Unit, IRCCS Sacro Cuore Don Calabria Hospital, 37024 Negrar di Valpolicella, Italy; 5 Sport Unit, IRCCS Sacro Cuore Don Calabria Hospital, 37024 Negrar di Valpolicella, Italy; 6 Medical Direction, IRCCS Sacro Cuore Don Calabria Hospital, 37024 Negrar di Valpolicella, Italy; 7 UOC Chirurgia Senologica, Ospedale Fatebenefratelli Isola Tiberina, Gemelli Isola, 00186 Rome, Italy; 8 UOC Oncologia Medica, Ospedale Fatebenefratelli Isola Tiberina, Gemelli Isola, 00186 Rome, Italy

**Keywords:** physical activity, risk level, breast cancer, quality of life, multidisciplinary

## Abstract

**Simple Summary:**

Operation Phalco is an experimental training protocol for breast cancer survivors implemented through a network of oncologists, sports medicine physicians and kinesiologists. The aim of this research is to evaluate the effects of an online adapted physical activity protocol with respect to a usual care on functional capacity, fatigue and quality of life of breast cancer patients. The protocol was structured by taking into account both cancer-related issues and the presence of comorbidities detected in the enrolled patients. For this reason, the patients recruited in the oncology centers of Rome and Verona were divided into three risk levels based on the risk stratification carried out by a sports medicine physician during a medical examination. The results evidenced the positive impact of a well-adapted physical activity on breast cancer patients’ quality of life, suggesting that this program should be included as early as possible in the cancer patient’s care process.

**Abstract:**

The international literature emphasizes the importance of physical activity (PA) in the first steps after cancer surgery. The regular practice of physical exercise causes positive adaptations on several functional capacities, with positive consequences on patients’ quality of life. This project aims to evaluate the effect of a post-operative training protocol, structured by taking into account both cancer-related issues and the presence of comorbidities, on functional capacities and quality of life of breast cancer survivors. Therefore, it was necessary to create a synergy between oncologists (referring physicians), sport medicine physicians (risk stratification and exercise prescription) and kinesiologists (trainers). Thirty-five post-surgery BC patients decided on a voluntary basis to attend an online Adapted PA (APA) protocol for 4 months, twice a week (APA Group) or Usual Care Group (UC Group). Functional capacity of the APA Group significantly increased, by 13.1% (*p* = 0.000), whereas perceived exertion decreased by 19.7% (*p* = 0.020). In the same group, the general health evaluated through the questionnaire EORTC-QLQ-C30 increased (*p* = 0.050). No differences were found in the UC Group. Operation Phalco, creating a network between oncologists, sports medicine physicians and kinesiologists, confirms the importance of structuring a post-operative path where APA should be included as early as possible in the cancer patient care.

## 1. Introduction

Physical activity (PA) is now recognized as an important tool for the prevention and treatment of breast cancer (BC), after diagnosis, and in the early steps after surgery [1]. Regular PA is related to the low incidence of eight different types of cancer [2] and it seems to have a protective and preventive role in the course of the disease [3,4]. Despite this knowledge, many cancer patients are not sufficiently active even though they express interest in PA, preferring instead to receive instructions from their oncologist on specific exercise activities [5]. Currently, the percentage of oncologists promoting exercise with their patients is still low, due to a lack of knowledge about PA and limited access to programs supervised by an exercise specialist [6]. To overcome these barriers, oncology care providers suggested introducing a kinesiologist as part of the clinical team to encourage exercise promotion [6]. An integrative approach promoting healthy lifestyle habits with PA in BC patients seems to have positive effects on quality of life (QoL) by better managing cancer symptoms rather than isolated interventions [7]. Furthermore, a multidisciplinary team makes it possible to identify comorbidities, according to which the type of PA may be proposed and the intensity of workloads can be best adapted. Adapted physical activity (APA) can induce several positive effects on the cardiovascular, respiratory, and musculoskeletal systems, inducing a positive impact on the patients’ QoL [8]. Studies evidenced that APA could protect the body against the stress related to treatments by mitigating their cardiotoxic effects, especially in BC patients [9]. During treatment, APA seems to increase the tolerance and enhance the therapies’ response to ameliorating the survival rate [3,4,10]. When it is proposed after treatment and it is integrated with the patient’s lifestyle, APA improves the BC patient’s QoL, reducing long-term side effects such as fatigue, pain, anxiety, stress, and the possibility of recurrences [11]. For these reasons, APA should be considered an integrative approach, alongside conventional cancer treatments, and should be included from the beginning of BC therapy. Despite this knowledge, there are still few BC patients engaged in PA programs [12]. Presumably, the lack of multidisciplinary interventions limits the dissemination and participation in regular PA of these patients. A cross-sectoral approach is suggested for BC patients but the difficulty of involving different professional figures still represents a barrier in the patient care process [13]. Another obstacle that limits the regular PA practice is the lack of individualized interventions based on a BC patient’s medical history and individual needs [14,15], as well as a clear methodology, based on FITT parameters (Frequency, Intensity, Time, Type), which is mandatory to adapt the exercise progression [16].

### Aim of the Study

The aim of this study was to evaluate the effectiveness of a multidisciplinary intervention (“PHALCO”—PHysicAL aCtivity for Oncology) that included oncologists, sport physicians, and kinesiologists on QoL and functional parameters in BC patients. More in-depth, well-tailored training was integrated during the post-operative BC period and the exercise protocol was administered and supervised online during the severe acute respiratory syndrome coronavirus 2 (SARS-CoV-2) pandemic emergency, providing the opportunity to develop a new online training strategy.

## 2. Materials and Methods

“PHALCO” is a multicenter Non Randomized Controlled Trial (mNRCT) developed in two parallel centers, San Giovanni Calibita Fatebenefratelli Isola Tiberina Hospital, Rome, and IRCCS Sacro Cuore Don Calabria di Negrar Hospital, in Verona. The institutional promoter was AIOM (Associazione Italiana di Oncologia Medica) Foundation, and the study coordinator was the University of Rome “Foro Italico”. The mNRCT was approved by the Ethical Committee Lazio 1 (RIF.232/CE) in agreement with the Declaration of Helsinki.

The study’s design was structured through a multidisciplinary approach where oncologists and breast surgeons recruited patients, the sports physicians provided risk stratification and exercise prescription, and the kinesiologist adapted and administered the exercise program. The intervention followed these mandatory steps:

Phase 1—Patient Enrolment

The oncologists/surgeons made an initial selection of BC patients according to the following inclusion criteria: women aged 40 to 65 years old (age range most represented in the two recruitment centers); physically inactive for at least 12 months for moderate-to-vigorous activities; women undergoing surgery (surgery performed at least 1 month prior and not more than 6); women subjected or not to radiotherapy (with completion of radiotherapy least 1 month prior and not more than 6) and adjuvant hormone therapy; in the case of breast reconstruction, the surgery had to be completed from at least 4 months; women undergoing adjuvant chemotherapy and/or radiotherapy, concluded at least 1 month prior and not more than 6.

We selected physically inactive but not sedentary women to avoid the need for a period of adjustment to the first exercise sessions. These women were not sedentary, but were engaged in light-intensity activity in their daily life. As suggested by Dempsey and colleagues, it is important to distinguish sedentary behavior from not engaging in physical activity, so as not to underestimate potentially cardiometabolic effects of light-intensity physical activity and the consequences of both physical activity and sedentary behavior on health outcomes [17].

Participants were excluded if they had metastatic disease at enrolment, musculoskeletal disturbances that can limit participation in the exercise training program, engagement in any formal exercise program for at least 12 months, or inability to give informed consent.

Phase 2—Risk stratification and division into levels and APA Prescription

At the beginning of the intervention (T0), the enrolled patients underwent a medical examination carried out by a sports medicine physician who performed the anamnesis to identify any risk factors and comorbidities of the BC patients, a 12 leads electrocardiogram (ECG) at rest and systemic arterial pressure analysis to give eligibility for the practice of recreational-motor activity. Based on risk stratification, participants were assigned, by the sport physician, three risk levels (Table 1) that are related to three levels of PA with different FITT parameters (Table 2), which have to be adapted, by the kinesiologist, according to the patient’s baseline functional parameters.

Phase 3—Functional and Psychological assessment

At the beginning of the study (T0) and at the end of intervention (T1), all patients were assessed by an APA kinesiologist on functional tests aimed to individualize an appropriate workload and to assess baseline fitness level (i.e., a 6 min walking test, 30 s sit-to-stand, handgrip, etc.). Moreover, the QoL and fatigue were assessed through specific questionnaires (EORTC-QLQ-C30 and EORTC-QLQ-FA12).

Phase 4—Training Planning and Adaptation

Based on the level assigned by the sports physician, APA kinesiologists, following the corresponding FITT prescription, adapted the training protocol based on the functional evaluations. BC patients performed a combined training (resistance and aerobic exercises) in two sessions per week for 4 months. Each training session lasted 60 min and was supervised by the kinesiologist online through Microsoft Teams video-calling. To guarantee safety and appropriate supervision, people enrolled in the exercise group were divided into small sub-groups (2–3 patients for each video-call). During the first online meeting, patients were educated to make the location where the training took place comfortable and safe. The kinesiologist made suggestions for setting up the microphone and video to avoid subsequent interruptions. Each participant was instructed regarding the use of the Borg scale (CR-10) and how to record heart rate with a personal smartwatch or manual pulse checking.

### 2.1. Sample Characteristics

A total of 35 post-surgery BC patients (40–65 years old), risk levels 1 and 2, were enrolled in the Rome and Verona centers. Before the beginning of the study, all patients provided written, informed consent. They were divided into two groups based on their preference: the exercise group (APA) and the Usual Care (UC) group. The APA group (*n* = 25) performed the well-tailored and adapted combined training program for 4 months, twice a week, online. The UC group (*n* = 10) followed usual care and was recommended to comply with current ACSM guidelines [18]. The BC women in the UC group were instructed to continue their usual activities. The ACSM guidelines [18] were explained individually by the APA kinesiologist to each patient during baseline assessments to promote awareness of the benefits of PA. The study did not include monitoring of PA levels in woman who followed usual care.

Most of the patients preferred to participate in the intervention group; for this reason, the two study groups are not perfectly balanced in number.

Patients’ characteristics are summarized in Table 3.

### 2.2. Functional and Psychological Assessments

Before (T0) and after (T1) 4 months of intervention, all patients underwent the following tests:

6-min walking test (6MWT), to evaluate functional capacity. Patients had to walk as far as possible (without running) for 6 min on a 30-m stretch of the corridor and the researcher recorded the walking distance and heart rate (HR). At the end of the test, the perceived exertion was evaluated through the BORG Scale (0–10) [19].

1-RM test using Brzycki’s formula [20] to assess lower extremity strength.

30 s sit-to-stand test to analyze functional strength of the lower limbs, transition movements, balance, and risk of falling [21].

The handgrip test to assess the muscle strength of both upper limbs using Jamar Plus^®^ dynamometer (Patterson Medical Ltd., Sutton-In-Ashfield, UK).

Trunk rotation and sit and reach test to test the flexibility [22].

Moreover, QoL and fatigue were evaluated through the European Organization for Research and Treatment of Cancer quality of life questionnaire (EORTC). Self-reported questionnaires EORTC-QLQ-C30 and EORTC-QLQ-FA12 were used to assess physical, emotional, and cognitive aspects or problems related to pathology and the general degree of fatigue. The psychometric coefficients were calculated with a sum score [23,24].

### 2.3. Training Protocol: Levels 1 and 2

Participants were divided into groups depending on the level assigned. The protocol lasted 4 months and was performed twice per week online using the Microsoft Team platform. Each session was divided into four phases:

Warm-up: lasted 10–15 min and it was focused on mobility and balance exercises. The mobility exercises involved neck, dorsal, upper limbs, and lower limbs mobilization, and they were aimed at gradually increasing the range of motion without inducing pain and respecting the patients’ individual abilities. Balance exercises were based on proprioceptive ability development through the conscious management of posture in static and dynamic positions.

Combined Training: resistance exercises: 30 min of resistance exercise for different muscle groups (upper limbs, core, back and lower limbs) were structured into a circuit modality. Depending on the risk level, this part was structured with body-weight exercises and/or exercises with dumbbells (1–3 kg). To set the baseline dumbbell weight, patients were assessed through the “biceps curl” exercise, where they performed 20 repetitions of biceps curl with a given dumbbell and provided a Borg scale value (CR-10). If the Borg value reported was between 2 and 4 during the test, the weight was appropriate for the training; if the Borg value was lower than 2, the weight was increased by 0.5 kg; and if the value was 4–6, the weight was decreased by 0.5 kg. The number of exercises, repetitions, and sets of resistance phase was increased every four weeks according to the Borg scale value provided by patients at the end of each circuit during the recovery. Aerobic exercises: patients were instructed about the combination and name of the aerobic workout based on different steps: lounge step, V step, leg curl, knee up, kick, punch, and merengue. They started to perform for 10 min and went up to 20 min at the end of the study. During the weeks, to increase the intensity, the use of the upper limbs was gradually introduced, and the rhythm of the musical bases was sped up (from 120 bpm to 130 bpm). Depending on the risk level, the work intensity was between 55–70% of the hearth rate reserve (HRR), calculated with the Karvonen formula (exercise target heart rate = [(220-age) − resting heart rate] × percent of exercise intensity + resting heart rate), and it was monitored by a heart rate pulse.

COOLDOWN: It included stretching exercises for the major muscle groups, with emphasis on the upper limbs. Each stretching position was kept for at least 20 s. Sometimes, according to the needs that emerged from dialogue with the patients, breathing and relaxation techniques were also included.

### 2.4. Statistical Analysis

The statistical package IBM SPSS, version 19 (Armonk, New York, NY, USA) was used for the analysis. Data were presented as mean values and standard deviations; the statistical significance was set at an alpha level of *p* < 0.05. The Shapiro–Wilk test was applied before the analysis to test the normal distribution of the data. When we obtained significant results, we conducted an interaction effect follow-up test by splitting the sample into two subgroups (i.e., training and control) and running separate repeated measures with ANOVA to explore the effect of time. The questionnaire data were analyzed using the ANOVA MIXED-way for repeated measurements. The total number of subjects needed to participate was estimated on the basis of a priori statistical power analysis. This analysis was computed using the G*Power software (G*Power V 3.1.3 Franz Faul, Universitat Kiel, Kiel, Germany), assuming a multivariate approach for intra-group effects (MANOVA for repeated measures). The following parameters have been considered for the procedure: the effect size for the main variable f = 0.33 (calculated from a partial frame age η^2^p = 0.10—mean effect); α = 0.05; and the correlation for repeated measures r = 0.5032. The estimated minimum number of patients required for the present study was 10 [25].

## 3. Results

This study enrolled a total of 34 subjects with BC after surgery. The mean age was 50.5 ± 5.7 for APA group and 45.1 ± 5.5 for UC group. A total of 16 patients had been subjected to quadrantectomy and 18 had been subjected to mastectomy. All of them concluded post-surgery treatment before starting training: 6 patients underwent chemotherapy + hormonotherapy + radiotherapy, 15 patients underwent hormonotherapy + radiotherapy, 11 patients underwent hormonotherapy, and 2 patients underwent immunotherapy.

After the first lesson, 1 patient was excluded from training due to the onset of a mild post-operative complication that led to an inability to train.

At the end of the study, there were no more drop-outs among the 34 remaining enrolled patients.

### 3.1. Functional Evaluation

Functional evaluations of the APA Group and UC group are reported, respectively, in Table 4 and Table 5. At the end of the training protocol, functional capacity, evaluated by the 6MWT, significantly increased by 13.1% (*p* = 0.000), whereas perceived exertion, assessed using the Borg scale, decreased by 19.7% (*p* = 0.020). UC group showed a reduction of 9.4% in 6MWT distance, and the perceived exertion increased by about 42%; these values do not reach statistical significance. The 30 s′ sit-to-stand test reported a significant improvement of 40.4% only in the APA group after training (*p* = 0,000) and no relevant changes were observed in the UC group. Lower limb strength measured with 1-RM leg press showed an improvement of 22.9% in the APA group (*p* = 0.040), while the UC group evidenced no differences from the baseline assessment. The handgrip data, however, revealed no significant strength changes in both UC and APA groups. Data evidenced an improvement in flexibility after training protocol. APA group reported an increase of 53.9% in sit and reach (*p* = 0.000), with an improvement of 14.2% on the right side (*p* = 0.010) and of 24.2% on left side, respectively (*p* = 0.000), on the trunk rotation test. UC group increased Sit and Reach value and slightly decreased Trunk Rotation tests; both changes did not achieve statistical significance. Regarding anthropometric measures, after APA intervention, weight was significantly reduced by 2.5% (*p* = 0.013) and BMI of 2.5% (*p* = 0.010). Again, no significant results were evidenced in the UC group.

### 3.2. Quality of Life and Fatigue Evaluation

The results of the EORTC QLQ-C30 and EORTC QLQ FA-12 questionnaires (Table 6) showed a positive trend in all items evaluated in the APA group. In particular, the items “Physical Function” reported a statistically significant increase of 3.3% (*p* = 0.050) and the “General Health” of 12.8% (*p* = 0.050). The “Emotional Fatigue” and “Interference with daily life” decreased, respectively, by 68.0% (*p* = 0.041) and 76.9% (0.019). As reported in Table 7, the UC group showed no significant changes.

## 4. Discussion

This study’s results showed that a multidisciplinary and integrated approach that includes oncologists, sport physicians, and APA kinesiologists is feasible and safe during the post-operative BC period, even in a pandemic situation, and it can improve QoL in these patients. Moreover, the APA group performed training sessions without adverse events or drop-out.

The effectiveness of synergy among all professional figures during intervention was demonstrated by the suspension of one patient from training. Due to the continuous supervision of the APA kinesiologist during the upper limb exercise execution, it was possible to detect discomfort early in one of the patients. After a revaluation by a sinologist surgeon, it was suggested not to continue the training. According to Misiag et al. (2022), it is extremely difficult to determine which type, intensity, and duration of physical activity may have the greatest effect on cancer patients; therefore, exercise should be individualized and based on the condition of the patient [26]. To overcome this limitation, the “PHALCO” project proposed a specific phase of intervention where sport physicians prescribe FITT parameters based on the patient’s clinical conditions and APA kinesiologists adapt the protocol according to the patient’s baseline characteristics. In this study, the risk stratification for clinical and cardiovascular complications has been performed. This methodology is suggested before the beginning and during the cancer treatment due to the high risk of cardiotoxicity development in BC patients [27]. In line with a recent consensus document on personalized exercise prescription for hypertension, the type and amount of exercise is prescribed not only according to age, gender, and ethnicity, but also according to comorbidities, personal preferences, and risk factors, such as baseline blood pressure levels [28]. Thus, the sport physicians’ prescription identified the PA range where the patients’ safety was ensured. Consequently, APA kinesiologists individualized and adapted the exercise considering both the level of physical condition and the patient’s clinical and cardiovascular risk.

Despite the positive aspects resulting from this collaboration among experts, adherence/compliance to APA protocols of BC survivors enrolled through this type of multidisciplinary approach will certainly have to be validated in subsequent studies, where it will be compared with a different type of patient recruitment.

In studies like this, it could be useful to implement the initial evaluation with bioimpedance as well, to better analyze how lean mass and fat mass can change with the practice of physical exercise. However, this was not the primary purpose of the study. It could be implemented in future projects to have a more complete view of the effects of physical exercise on the body composition of these patients.

Our protocol, in line with Schutz et al.’s, reported positive physiological and psychological effects in BC survivors, suggesting that this approach could overcome the PA barriers related to a lack of individualization [29,30]. Further in-depth, the results of our study evidenced an improvement in functional capacity assessed by 6MWT, which is an indicator of general health in BC patients [19], and it showed a reduction in fatigue perception during the test, indicating an improvement in the patient’s health status. The deconditioning of skeletal muscles is one of the main BC side effects [31]. The APA group increased the load lifted in the 1RM leg press and the number of repetitions performed during the 30″ STS test; these data indicate an increased muscle strength, which could be a general indicator of functional lower limb strength and balance improvement [21]. Otherwise, the assessment of upper limbs’ strength did not provide any significant changes in APA; this could be due to the limited overloads used during the 4-month intervention (1–3 kg), especially because the exercise protocol was administered by video-call, and it was necessary to ensure that the exercises were performed safely. Moreover, “PHALCO” training was structured to recover the function of the operated limb, not to improve the strength. In line with Mirandola et al. (2020), the data from Trunk Rotation test suggest that an adapted PA intervention may represent an effective strategy to improve the efficiency of the injured arm in BC patients [32]. According to the studies reported in the literature, the psychological results from the EORTC-QLQ-C30 and EORTC-QLQ-FA12 questionnaires showed a positive trend of all items analyzed after APA intervention, indicating a positive effect on QoL and general health perception in BC patients [33,34]. Particularly, only the APA group showed an improvement in “Interference with daily life” and “Physical functioning” items, underlying that APA should be considered as an integrative therapy to the conventional clinical treatments for BC patients. According to previous studies, “PHALCO” results implemented data about the sustainability and feasibility of online training supervised by kinesiologists in BC patients [34,35].

Despite these positive results, the study had some limitations: the difference in the number of participants between the group, which does not allow a proper data comparison between APA and UC groups. Moreover, no patients with risk level 3 were enrolled in the study; this is probably due to the difficulties in involving BC patients with different comorbidities in well-tailored physical activity.

## 5. Conclusions

The “PHALCO” protocol seems to be efficient and effective as a multidisciplinary approach in oncology where an APA protocol is integrated. The synergy between oncologists/sinologists, sport physicians, and APA kinesiologists could be an effective strategy to increase the participation of BC patients in exercise protocols. Our study confirms that many women with a history of BC are interested in practicing an APA and that, if they feel followed and safe, they complete the training protocol, reducing the risk of dropping out. The standardization of each intervention phase makes this intervention reliable, easily reproducible, and safe. “PHALCO” suggests different levels of exercise with defined FITT parameters according to both the physical condition and comorbidities of the BC patient. This framework could be a starting point for new therapeutical strategies based on patient characteristics, which would allow us to detail even more accurately which is the most appropriate exercise for each individual patient. Combined training was confirmed as improving QoL, functional capacity, and muscle strength and reducing the perception of fatigue, and seems to be the best APA protocol able to support the standard treatment; therefore, it should be included as early as possible in the rehabilitation process of the BC patient.

## Figures and Tables

**Table 1:** Risk levels based on patients’ comorbidities.

Level 1	Level 2	Level 3
BC patients without severe comorbidities	BC patients with two or more of the following risk factors: Hysterectomy or postmenopausal Smoker (or who has quit smoking for less than 6 months) Blood pressure >140/90 mmHg Dyslipidaemia Overweight Positive family history for heart attack or intervention for a cardiovascular disease before age 55 (father or brother) or age 65 (mother or sister)	BC Patient with at least 1 of the following risk factors: Cardiovascular disease (ischemic heart disease, valve disease, etc.) Diabetes Asthma Osteoporosis Musculoskeletal disorders

**Table 2:** FITT (Frequencies, Intensity, Time, Type) prescription according to the risk levels. Abbreviation: 1RM = one Repetition Maximum; HRR = Heart Rate Reserve; min = minutes.

FITT Level 1	FITT Level 2	FITT Level 3
Warm-up: about 10 min	Warm-up: about 10 min	Warm-up: about 10 min
Combined Training:	Combined Training:	Combined Training:
Resistance training: 2 sets of 8 repetitions at 60–70% 1 RM—about 25 min	Resistance training: 2 sets of 8 repetitions at 50–60% 1 RM—about 25 min	Resistance training: 2 sets of 8 repetitions at 10–20% 1 RM—about 25 min
Aerobic training: 65–70% of HRR—about 15 min	Aerobic training: 60–65% of HRRM—about 15 min	Aerobic training: 55–60% of HRRM—about 15 min
Cool Down: Stretching exercises for large muscle groups—10 min	Cool Down: Stretching exercises for large muscle groups—10 min	Cool Down: Stretching exercises for large muscle groups—10 min

**Table 3:** Patients’ characteristics. Abbreviation: APA = Adapted Physical Activity; UC = Usual Care M = mean; SD = Standard Deviation; *n* = numbers; BMI = Body Mass Index; Chemo = Chemotherapy; Hormo = Hormonal therapy; Radio = Radiotherapy; Immuno = Immunological therapy.

Patients’ Characteristics	APA Group *n* = 24 (M ± SD)	UC *n* = 10 (M ± SD)
Age (years)	50.5 ± 5.7	45.1 ± 5.5
Weight (kg)	67.0 ± 14.0	55.3 ± 1
High (cm)	164.1 ± 5.8	157.5 ± 3.5
BMI (kg/m^2^)	24.7 ± 4.1	21.3 ± 0.5
Type of intervention		
Quadrantectomy	10	6
Mastectomy	14	4
Treatments		
Chemo + Hormo + Radio	4	2
Hormo + Radio	10	5
Hormo	8	3
Chemo + Immuno	2	0

**Table 4:** Pre- and post-functional evaluation APA group. Abbreviation: M = Median; IQR = Interquartile Range Diff = Differences in percentage; *n* = numbers; BMI = Body Mass Index; 6MWT = Six min walking test; 1-RM = One Repetition Maximum; R = right; L = left; n.s. = no statistical significance.

APA Group *n* = 24	Pre-Training M (IQR)	Post-Training M (IQR)	Diff Post/Pre (%)	*p*-Value
Weight (kg)	63.0 (18.0)	61.9 (18.1)	−2.5	0.013
BMI (kg/m^2^)	23.7 (5.9)	23.3 (3.9)	−2.5	0.010
6MWT (m)	531.9 (151)	588 (89)	+13.1	0.000
BORG (0–10)	2.5 (1.8)	2.0 (1.4)	−19.7	0.020
1-RM Leg Press (kg)	113.9 (43.3)	160.8 (58.3)	+22.9	0.040
Handgrip R (kg)	27.8 (3.8)	31.0 (4.8)	+8.6	n.s.
Handgrip L (kg)	26.5 (2.8)	28.7 (7.0)	+4.1	n.s.
30′ Sit to Stand (*n*)	18.5 (12)	25.5 (8)	+40.4	0.000
Sit and Reach (cm)	8.2 (6.9)	11 (9.1)	+53.9	0.000
Trunk Rotation R(cm)	63 (17.1)	69.5 (33.3)	+14.2	0.010
Trunk Rotation L (cm)	58.5 (27.5)	67.0 (37.5)	+24.2	0.000

**Table 5:** Pre- and post-Functional evaluation UC group. Abbreviation: M = Median; IQR = Interquartile Range; Diff = Differences in percentage; *n* = numbers; BMI = Body Mass Index; 6MWT = Six min walking test; 1-RM = One Repetition Maximum; R = right; L = left; n.s. = no statistical significance.

UC Group *n* = 10	Pre M (IQR)	Post M (IQR)	Diff Post/Pre (%)	*p*-Value
Weight (kg)	56.5 (9.9)	54 (16.4)	+4.5	n.s.
BMI (kg/m^2^)	22 (1.4)	22.9 (1)	+6.5	n.s.
6MWT (m)	525 (55)	505 (125)	−9.4	n.s.
BORG (0–10)	1.0 (1)	2 (2)	+42	n.s.
1-RM Leg Press (kg)	91.0 (33)	93.1 (39)	+1.5	n.s.
Handgrip R (kg)	29.4 (5.3)	29.4 (9.3)	−3.9	n.s.
Handgrip L (kg)	24.5 (2.8)	20.9 (0.8)	−13.5	n.s.
30′ Sit to Stand (*n*)	20.0 (8.0)	20.0 (8.0)	−5.2	n.s.
Sit and Reach (cm)	5 (23)	4 (23)	+57.5	n.s.
Trunk Rotation R (cm)	42.0 (6)	35.0 (8)	−4.3	n.s.
Trunk Rotation L (cm)	33 (9)	43 (9)	−4.0	n.s.

**Table 6:** Pre- and post-EORTC QLQ C-30 and EORTC QLQ FA-12 analysis—APA Group. Abbreviation: M = Median; IQR = Interquartile Range; Diff = Differences in percentage; *n* = numbers; WDL = with daily life; n.s. = no statistical significance.

EORTC QLQ C-30				
APA Group *n* = 24	Pre-Training M (IQR)	Post-Training M (IQR)	Diff Post/Pre (%)	*p*-Value
Physical Function	93.3 (6.6)	93.3 (11.6)	+3.3	0.050
Emotional Function	83.3 (31.2)	95.9 (16.6)	+8.1	n.s.
Cognitive Function	100 (16.6)	100 (12.5)	+0.8	n.s.
Social Function	100 (33.3)	100 (12.5)	+9.9	n.s.
Global Health	75.0 (33.3)	83.3 (14.5)	+12.8	0.050
**EORTC QLQ FA-12**				
Physical Fatigue	20.0 (23.3)	6.66 (13.3)	−32.6	n.s.
Emotional Fatigue	0.0 (22.2)	0.0 (0.0)	−68.0	0.041
Cognitive Fatigue	0.0 (12.7)	0.0 (0.0)	−66.7	n.s.
Interference WDL	33.3 (33.3)	0.0 (0.0)	−76.9	0.019

**Table 7:** Pre- and post-EORTC QLQ C-30 and EORTC QLQ FA-12 analysis—UC Group. Abbreviation: M = Median; IQR = Interquartile Range; Diff = Differences in percentage; *n* = numbers; WDL = with daily life; n.s. = no statistical significance.

EORTC QLQ C-30				
UC Group *n* = 10	Pre M (IQR)	Post M (IQR)	Diff Post/Pre (%)	*p*-Value
Physical Function	93.3 (6.6)	93.3 (6.6)	+1.1	n.s.
Emotional Function	91.6 (16.6)	91.6 (8.3)	−0.7	n.s.
Cognitive Function	100 (16.1)	83.3(16.6)	−2.7	n.s.
Social Function	100 (33.3)	83.3 (33.3)	−2.7	n.s.
Global Health	75.0 (41.7)	66.7 (33.3)	−10.7	n.s.
**EORTC QLQ FA-12**				
Physical Fatigue	13.3 (13.3)	13.3 (13.3)	+7.1	n.s.
Emotional Fatigue	0.0 (22.2)	0.0 (22.2)	+16.6	n.s.
Cognitive Fatigue	0.0 (0.0)	0.0 (22.2)	+40.0	n.s.
Interference WDL	33.3 (33.3)	33.3 (31.3)	+19.7	n.s.

## Data Availability

Data supporting the reported results can be obtained from the corresponding author.
